# Grant Report on Social Reward Learning in Schizophrenia ^†^

**DOI:** 10.20900/jpbs.20200004

**Published:** 2020-02-27

**Authors:** Pamela D. Butler, Matthew J. Hoptman, David V. Smith, Julia A. Ermel, Daniel J. Calderone, Sang Han Lee, Deanna M. Barch

**Affiliations:** 1 Nathan Kline Institute for Psychiatric Research, Orangeburg, NY 10962, USA; 2 Department of Psychiatry, New York University School of Medicine, New York, NY 10016, USA; 3 Department of Psychology, Temple University, Philadelphia, PA 19122, USA; 4 Department of Child and Adolescent Psychiatry, New York University School of Medicine, New York, NY 10016, USA; 5 Departments of Psychological & Brain Sciences, Psychiatry & Radiology, Washington University, St. Louis, MO 63130, USA

**Keywords:** schizophrenia, reward, social cognition, ventromedial prefrontal cortex, striatum, amygdala, expected value, prediction error

## Abstract

We report on the ongoing R21 project “Social Reward Learning in Schizophrenia”. Impairments in social cognition are a hallmark of schizophrenia. However, little work has been done on social reward learning deficits in schizophrenia. The overall goal of the project is to assess social reward learning in schizophrenia. A probabilistic reward learning (PRL) task is being used in the MRI scanner to evaluate reward learning to negative and positive social feedback. Monetary reward learning is used as a comparison to assess specificity. Behavioral outcomes and brain areas, included those involved in reward, are assessed in patients with schizophrenia or schizoaffective disorder and controls. It is also critical to determine whether decreased *expected value* (EV) of social stimuli and/or *reward prediction error* (RPE) learning underlie social reward learning deficits to inform potential treatment pathways. Our central hypothesis is that the pattern of social learning deficits is an extension of a more general reward learning impairment in schizophrenia and that social reward learning deficits critically contribute to deficits in social motivation and pleasure. We hypothesize that people with schizophrenia will show impaired behavioral social reward learning compared to controls, as well as decreased ventromedial prefrontal cortex (vmPFC) EV signaling at time of choice and decreased striatal RPE signaling at time of outcome, with potentially greater impairment to positive than negative feedback. The grant is in its second year. It is hoped that this innovative approach may lead to novel and more targeted treatment approaches for social cognitive impairments, using cognitive remediation and/or brain stimulation.

## SIGNIFICANCE

Schizophrenia affects ~1% of the population and takes a remarkable toll on patients and families, often leading to lifelong disability. Deficits in social cognition are a hallmark of this disorder [1,2]. Social cognition refers to the “mental operations that underlie social interactions….” [3] (p. 1211) and are linked to poor community functioning [4] and functional disability in schizophrenia [5,6]. Some of the social cognitive impairments that have been identified in schizophrenia include deficits in social cue identification, empathy, theory of mind, and emotion regulation [1,2]. Indeed, these deficits have a greater negative impact on functioning than non-social cognition [4]. People with schizophrenia show social anhedonia and profound disinterest and lack of engagement in social interactions [7–10]. The rewarding nature of social feedback and social interactions has received growing attention in neuroscience research [11–15]. Humans are motivated to engage in social behavior, and pro-social behavior has a rewarding quality similar to other types of rewards [11,12]. Thus, impaired ability to learn from social rewards could greatly impact the ability and motivation to interact successfully with others. Despite the profound social cognitive deficits and the lack of engagement in social interactions in this disorder, to our knowledge there is relatively little work on social reward learning in schizophrenia [16–19] although there is robust evidence for impairment in non-social (e.g., monetary) reward learning in schizophrenia. Given the growing knowledge about social reward learning in neuroscience research, the time is ripe for investigating social reward learning in schizophrenia. The crux of this research grant is to fill this gap in the literature by studying behavioral manifestations and neural underpinnings of social reward learning deficits in schizophrenia, assessing relationships between social reward learning and impaired motivation and pleasure, and assessing the specificity of any such relationships to social versus monetary reward learning.

### Reward Circuitry

**Figure 1.**
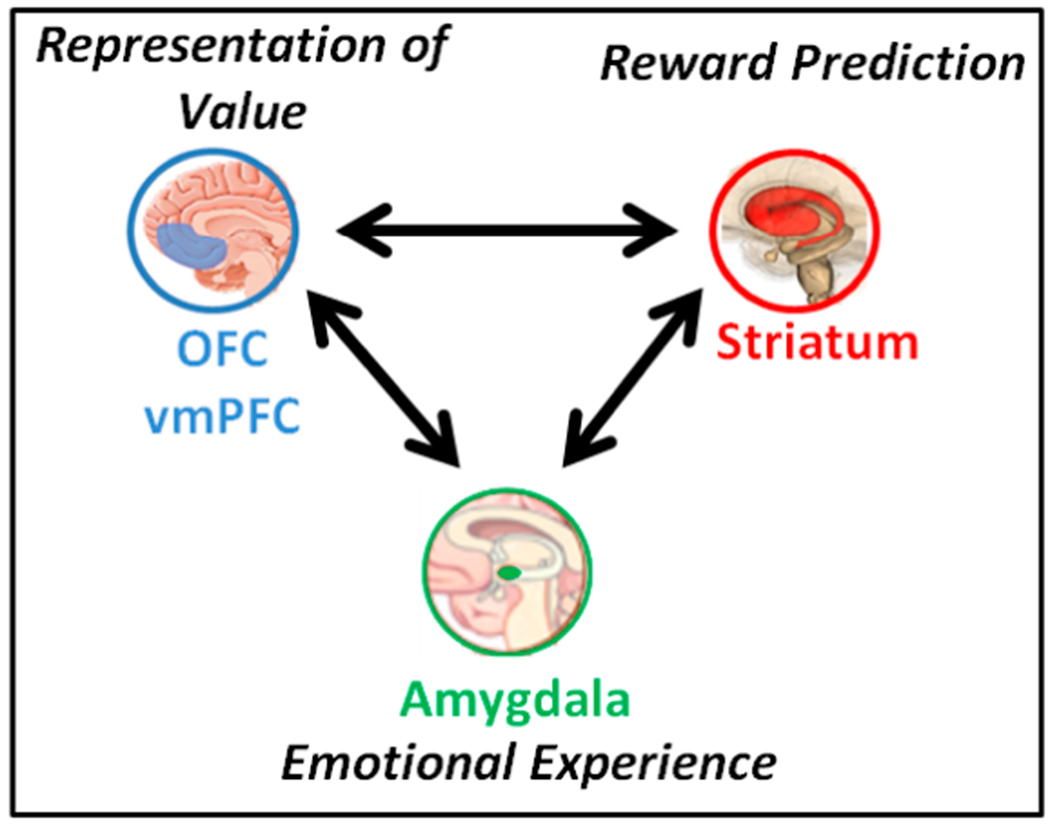
Schematic of brain areas thought to be involved in social reward learning and their function.

The cortical-basal ganglia circuit is critical to reward processing and decision making [20]. Two fundamental processes of this reward circuit that are relevant for this grant project are reward prediction errors (RPE) and the representation of expected value (EV) [21] (Figure 1). Dopamine projections from the ventral tegmental area to the striatum are particularly important for reward learning [20]. These neurons code RPEs which occur at the time an outcome is received. Positive RPEs are associated with a phasic increase in dopamine firing when the outcome is better than expected and are thought to reinforce associations [22]. Negative RPEs are associated with a decrease in phasic dopamine firing when the outcome is worse than expected and are thought to weaken associations. RPEs and striatal activity are thought to represent implicit reward learning (e.g., learning that is not necessarily in conscious awareness) [23–25]. EV comes into play at the time of choice when one has to compare different options in order to make a choice [26,27]. This involves the orbitofrontal cortex (OFC) and more specifically the ventromedial prefrontal cortex (vmPFC) [15,28]. The generation of EVs by prefrontal cortex is thought to represent explicit reward learning (e.g., conscious learning that is under cortical control) [25,29–32].

Much of the work regarding RPE and EV was initially done with nonsocial rewards such as money and juice, but more recently the effects of social rewards have been studied [11–15]. As would be expected, some of the same basic reward circuitry is also involved in social reward learning. For instance, social and non-social reward were found to have a “common currency”, such that the vmPFC encodes information about the decision value of money and decision value of faces in an economic exchange task [13]. In addition, in parallel social and monetary probabilistic reward learning (PRL) tasks [14,33], similar areas were activated in the vmPFC modulated by EV at time of choice and in the striatum modulated by RPE at time of outcome. These parallel tasks are being used in the present study. Similarly, neural representations of social rewards involving acquiring a good reputation activated the same area of the left striatum as monetary reward [34]. Indeed, a recent meta-analysis of human fMRI studies found that there is a common brain area—the vmPFC—that represents the EV of different types of stimuli, including social rewards [15]. Thus, some of the same basic reward circuitry seen in nonsocial reward studies is also involved in social reward. However, a number of other brain areas are more specifically involved in social cognition and social reward learning, including the amygdala, which responds to the emotional valence of stimuli, including faces [1,35–37]. Altered amygdala functioning in schizophrenia [1,35,36,38,39] may be involved in social reward learning deficits, as this area projects to the striatum and PFC, such that decreased amygdala responses to the valence of social stimuli could affect RPE learning as well as the valuation of stimuli (Figure 1). One of the few social reward studies we are aware of in schizophrenia that have looked at brain function used a social reputation task and found decreased parietal lobe activation and a significant relationship between subjective ratings of positive feedback and right insula activation in patients [40], though this task did not involve learning. In addition, Fett et al. [17] found aberrant caudate and right temporoparietal junction (TPJ) responses in people with schizophrenia in a trust game that involved elements of social reward (e.g., learning the intentions of others).

### Reward Learning in Schizophrenia

Consistent deficits in monetary reward learning are seen in this disorder [25,31,41–44], which are related to decreased motivation and pleasure [25,44–48]. Results from behavioral monetary reward learning studies may inform hypotheses about social reward learning since key reward areas such as the vmPFC and striatum respond similarly to both types of rewards in controls [13–15,49] and similar responses to social and non-social stimuli are seen in some behavioral studies in schizophrenia (e.g., ratings of consummatory pleasure) [50,51]. In schizophrenia, monetary reward learning deficits in many, but not all, studies show impaired learning to positive feedback, but relatively intact learning to avoid negative feedback [25,31,41,43,46,52–55]. The proposed social reward learning studies may provide similar evidence of impaired learning to positive, but more intact learning to negative, social feedback. If people fail to learn from positive social experiences, but do learn from negative experiences, this would cause a “perfect neurobehavioral recipe” for decreased social motivation and pleasure, similar to what has been described for non-social learning [21]. It is also critical to determine whether decreased EV of social stimuli and/or RPE learning underlie social reinforcement deficits so as to inform potential treatment pathways. Impaired monetary reward learning in schizophrenia is driven in part by decreased value of rewards. This is seen in computational modeling studies of reward learning behavior in schizophrenia [27,31,44] as well as in decreased activation of vmPFC and other frontal cortical areas at time of choice in schizophrenia [27,41,42,56], including less frontal activation to positive feedback in patients with greater anhedonia/avolition [41]. In addition, people with schizophrenia fail to employ appropriate win/stay and lose/shift strategies, which reflect difficulty in trial to trial updating of value [27,43,46,57]. However, studies of striatal RPE signaling in schizophrenia are more mixed. Decreased striatal activation to RPEs is seen in some studies [48,58–60] and, like the behavioral results, is observed with positive, not negative RPEs [61,62]. Yet, not all studies in schizophrenia show decreased striatal RPE activity [41] and medication may be a factor [63,64]. The stage of learning is also important, with greater decreases in activation of brain reward areas early in learning at time of choice in patients compared to controls [41]. If impaired social reward learning is driven by decreased ability to generate EV of stimuli, this would indicate more of a cortical control/explicit reward learning deficit. In contrast, if impaired social reward learning is driven by decreased striatal RPE activity, this would indicate more of an implicit deficit that may be out of conscious awareness.

The PRL paradigm we are using to assess social reward learning in schizophrenia was designed by Lin and colleagues [14,33] and has a number of appealing aspects including parallel social and monetary reward learning versions, assessment of learning to gain as well as to avoid loss, and the ability to model vmPFC function by EV and striatal function by RPE. In addition, this paradigm was successfully used in an autism study showing greater deficits to social than monetary reward learning versus controls [30]. As described in the APPROACH Section below, we modified this task for use in schizophrenia.

Several previous studies have looked at other aspects of social reward learning in schizophrenia, mainly using a “trust game” in which the participant (“investor”) is given money and has to decide whether to share this with a computer-simulated partner who may or may not repay some of the money to the investor [17,65,66]. The investor is told that if the partner repays some of the money, it will be more than the initial investment. There are usually a number of rounds so that the investor has the opportunity to learn whether the confederate is trustworthy (i.e., gives money back to the investor). The trust game differs from the PRL task as the former utilizes theory of mind and thus activates medial prefrontal cortex (mPFC) and TPJ [17,67–69]. Of areas involved in reward processing, the trust game activates striatum [65,68–72]. Thus, the trust game and PRL task (see Reward Circuitry Section above) activate somewhat different circuitry, though they overlap in striatal activation. In addition, unlike PRL tasks [27,31,44] reward parameters of the trust game, including a social value model, have only been modeled in a few studies with controls [66,68], and have not been modeled in studies of people with schizophrenia. However, results from trust game studies may inform hypotheses of the current study. Hanssen et al. [19] compared social (trust) to non-social (lottery) game reward tasks in schizophrenia, and found impairment in both tasks in patients compared to controls, providing support for our hypothesis that social reward learning will be an extension of more general reward learning impairments in schizophrenia. In addition, Campellone et al. [18] modified the trust game to investigate responses to positive and negative rewards. They reported that people with recent onset schizophrenia spectrum disorders placed less trust in others and expended less effort to have more positive social interactions, but not negative social interactions, compared to controls. These social results are consistent with previous monetary reward learning studies in schizophrenia that have found evidence of greater impairments for learning about positive versus negative outcome [25,31,41,43,46,52–55] and further support the present hypothesis that social reward learning on the PRL task may be more impaired to positive than negative feedback. A parallel monetary reward learning task was not, however, used in this modified trust game study by Campellone et al. [18].

Several recent studies have looked at effects of remediation on social reward processing in schizophrenia [73,74]. Vinogradov and colleagues paired computerized social cognitive training with “cold” cognitive training [73,74] because of previous studies suggesting that poor social cognitive abilities may impede motivation and lead to poor functioning [73]. Greater improvements in both cold cognition (e.g., prosody identification) and self-reported reward processing were found following the combined treatment than following cognitive treatment alone [73] which were sustained at six-month follow up [74]. Other groups have also found greater improvements with combined treatments [75]. These studies suggest reward processing can be improved by remediation. However, psychometrically validated social reward learning tasks have yet, to our knowledge, to be developed for use in clinical trials. The modified trust game developed by Campellone and colleagues [18], described above, was used to assess effects of a motivation-enhancing remediation [76]. Improvements were found in several aspects of motivated behavior but not in social reward (e.g., trust) behavior itself [76]. Knowledge about social reward learning from the current grant project and from other studies [18,19] may pave the way to providing information that will be useful in development of remediations targeted at social reward learning deficits and in development of psychometrically validated social reward learning tasks to be used as outcome measures.

As sometimes happens, a similar study was published after we began our grant project. Lee and colleagues [16] looked at social vs. monetary reward learning in schizophrenia also using the same paradigm from Lin and colleagues [14,33] that we are using. Lee et al. [16] reported that controls showed similar activations on the social and monetary tasks in ventral striatum, vmPFC, and anterior cingulate cortex ROIs. Schizophrenia patients showed significantly decreased activations in these areas on the social, but not the monetary task, compared to controls. However, Lee et al. [16] showed brain activations only for the contrast of gain versus avoid loss trials, so that it was not clear whether decreases in the gain condition, increases in the loss condition, or some combination were driving the results. Interestingly, there were no between-group differences in reward learning behavior on either the social or monetary task, but both groups performed better on the money than social task. However, statistics, but not behavioral data itself, were presented so it is unclear how participants did on the task. No significant relationships were found between reward learning and symptom ratings. The findings of Lee et al. [16] are not in line with our hypothesis that behavioral and brain activation impairments will be seen in patients in both types of reward learning (see Central Hypotheses below) or with a number [25,31,41–43], though not all [63] previous studies showing behavioral and/or MRI reward learning deficits to non-social stimuli. However, in the Lee et al. [16] study, performance was overall better for controls in the monetary versus social learning task, making direct comparisons difficult. Further, Lee et al. [16] did not distinguish between choices that involved earning reward versus those that involved avoiding loss in their behavioral analysis. However, a recent trust game study described above reported results more in line with our hypotheses, showing impairments in both social and non-social reward learning in schizophrenia spectrum individuals [19]. For the current study, we made a number of modifications to Lin et al. [14,33] task that we believe allow for a clearer test of our hypotheses. Specifically, we optimized the behavioral aspects of the task so that controls do equally well on both tasks, a critical psychometric characteristic necessary to establish differential deficits in a patient group. Further, these modifications ensure that patients are able to learn the task to a criterion significantly better than chance (see APPROACH Section). As the Lee et al. [16] study is the first published work, to our knowledge, to directly assess social reward learning using a PRL task in schizophrenia, replication is extremely important.

We anticipate that our findings will extend those of the two previous studies that assessed parallel social and non-social reward learning in schizophrenia [16,19] by our novel goals of separately assessing learning to positive and negative stimuli and by looking at modulation of brain reward areas by EV and RPE to further understand mechanisms of deficits and their relative relationships to social impairments and anhedonia in schizophrenia.

### Dysconnectivity

“Dysconnectivity” is an influential theory regarding the pathophysiology of schizophrenia [77]. Impaired resting state functional connectivity involving the nucleus accumbens and default mode network was recently found to be related to reward deficits in schizophrenia [78], though relationships between functional connectivity and reward-related areas have not been widely studied [79]. An exploratory analysis will assess resting state functional connectivity between three sets of areas that we hypothesize might show impaired connectivity related to social reward learning in schizophrenia (prefrontal cortex/striatum, prefrontal cortex/amygdala, and amygdala/striatum). Numerous schizophrenia studies suggest impaired frontal/striatal interactions [43,80,81] including strength of resting state OFC/striatal connectivity that was related to decreased motivation [82], and it has been suggested that altered value signaling in vmPFC may contribute to impaired striatal RPE responses in schizophrenia [27,56]. Thus, decreased prefrontal/striatal connectivity may be related to impaired striatal RPE signaling to social reward outcome. The amygdala has a number of connections throughout the brain including with classic mPFC and striatal reward areas [20,83]. Due to altered amygdala activation in schizophrenia [35,36,38] and altered connectivity between the amygdala and mPFC seen in psychophysiological interaction studies [84–86], we hypothesize that decreased amygdala connectivity with mPFC will be related to decreased vmPFC EV signaling at choice and that decreased amygdala connectivity with striatum will be related to decreased striatal RPE signaling at outcome. We also hypothesize that altered vmPFC/striatal and vmPFC/amygdala connectivity will be related to impaired social motivation.

## CENTRAL HYPOTHESES AND SPECIFIC AIMS

Our central hypothesis is that the pattern of social learning deficits in schizophrenia is an extension of more general reward learning impairments in schizophrenia and that social reward learning critically contributes to deficits in social motivation and pleasure. Thus, we focus on classic reward areas and look at striatal RPE signaling at time of outcome and vmPFC EV signaling at time of choice. Similar to what is seen in monetary studies, we hypothesize decreased signaling in both areas in patients compared to controls. However, crucially, there may be additional impairments in brain areas related to social cognition and it is possible that impairments in social reward learning are more strongly related to functional impairments in social relationships than are deficits in non-social reward learning. Thus, it is also possible that the neural correlates of impaired social reward learning may also differ in important ways from non-social reward learning, such that regions such as the amygdala that are involved in social cognition will contribute to social reward learning impairments. We will assess amygdala activation at outcome since it responds to valence of faces. Monetary reward learning will be used as a comparison to assess specificity.

The Specific Aims are:

### Aim 1: Assess whether people with schizophrenia show impaired learning from social rewards.

H1: People with schizophrenia will show impaired social reward learning, with potentially greater impairment to positive, than negative, social feedback.

### Aim 2: Evaluate neural circuitry involved in social reward learning in schizophrenia.

H2: Patients with schizophrenia will show decreased vmPFC EV signaling at time of choice and decreased striatal RPE signaling at time of outcome, with greater decreases earlier in learning, and potentially greater impairment to positive than negative feedback; H3: Patients will show impaired amygdala activation at time of reward; H4: An exploratory goal is to assess functional connectivity between vmPFC and striatum, mPFC and amygdala, and between amygdala and striatum.

### Aim 3: Assess relationships between social and monetary reward learning and clinical symptoms and function in schizophrenia.

H5: Social reward learning deficits will be related to decreased motivation/pleasure and to impaired function. H6: A secondary hypothesis is that social reward learning deficits will be related to impaired social motivation/pleasure and social function whereas monetary reward learning deficits will be related to impaired non-social motivation/pleasure and non-social function.

## INNOVATION

The grant project is innovative on a number of levels. While much research has been done on social cognitive deficits in schizophrenia [1], little work has been done to assess social reward learning in schizophrenia, with the exception of several recent papers [16–19] or to assess parallel social and non-social reward learning in schizophrenia [16,19]. As detailed above, a number of studies have assessed monetary reward learning in schizophrenia, which have provided important information regarding decision making and underpinnings of decreased motivation and pleasure [25,45–48]. The goals of assessing learning to negative and positive social feedback, using modeling to assess impairments in RPE and EV, including EV signaling in the vmPFC at time of choice and RPE signaling in the striatum at time of outcome, as well as function of amygdala, which is important in assessing social valence, at time of outcome are all novel. Assessment of the relationships between behavioral and brain responses to social reward learning in relation to motivation and pleasure, areas that people with schizophrenia show impairment in and that are related to poor function, has been done in only a few studies [16,18]. In addition, the exploratory aim of looking at resting state functional connectivity has not, to our knowledge, been assessed in schizophrenia between mPFC and amygdala or between amygdala and striatum, though studies have utilized task-based measures including psychophysiological interactions to look at relationships between mPFC and amygdala [84,85]. Assessing relationships between resting state functional connectivity and social reward processing measures is also novel. Finally, it is hoped that this innovative approach to understanding social cognitive difficulties, which are likely to bear on social interactions, may lead to novel and more targeted treatment approaches for social cognitive impairments, using cognitive remediation and/or brain stimulation (see SUMMARY AND FUTURE DIRECTIONS Section).

## APPROACH

### Overall Project Structure

This is an R21 that is in its second year. Dr. Pamela Butler is the Principal Investigator. There is one site. Participants are recruited and receive behavioral and MRI testing at the Nathan Kline Institute for Psychiatric Research (NKI). Ms. Julia Ermel is the project coordinator at NKI. Dr. Matthew Hoptman oversees the fMRI analysis and functional connectivity analyses. Dr. Daniel Calderone is responsible for carrying out the MRI analyses. Dr. Sang Han Lee, at NKI, is the statistical analyst. Dr. David Smith at Temple University is a consultant and performs the computational modeling and consults on analyses and interpretation of data. Dr. Barch at Washington University is a consultant and provides overall guidance on the paradigm, analysis, and interpretation of data. All personnel are involved in manuscript preparation.

Dr. Butler has expertise is in perceptual, cognitive, and social cognitive function in schizophrenia utilizing psychophysical, electrophysiological, and MRI techniques. This grant project continues her long-standing collaboration with Dr. Matthew Hoptman at NKI, who is an expert on MRI in schizophrenia, including task-based and resting state functional connectivity. She has developed new collaborations with Dr. Deanna Barch, who has expertise in behavioral and neuroimaging paradigms relevant to cognition, motivation, and reward, and with Dr. David Smith, whose specialties are neuroimaging of social and non-social reward processing, and computational modeling of reward learning. Dr. Butler worked closely with them to develop this project. Other personnel include Dr. Lee who has extensive experience performing statistical analyses for behavioral psychiatric research as well as molecular and cellular neurobiology, and neuroimaging-based projects. Dr. Calderone did his dissertation work with Dr. Butler and has expertise in electrophysiology and MRI analyses, including functional and resting state MRI. In addition, Ms. Ermel did undergraduate research in Dr. Barch’s lab and brings experience in working with people with schizophrenia and understanding of motivation and social function to her position as project coordinator.

### Study Design

Patients with schizophrenia and controls will participate. Participants will receive a diagnostic interview (Structured Clinical Interview for Diagnosis), clinical ratings, questionnaires, medication history, and demographic history. The PRL social and monetary tasks are performed in the MRI scanner and resting state scans are also be obtained.

### Reward Learning and Face Emotion Assessment Tasks

**Figure 2.**
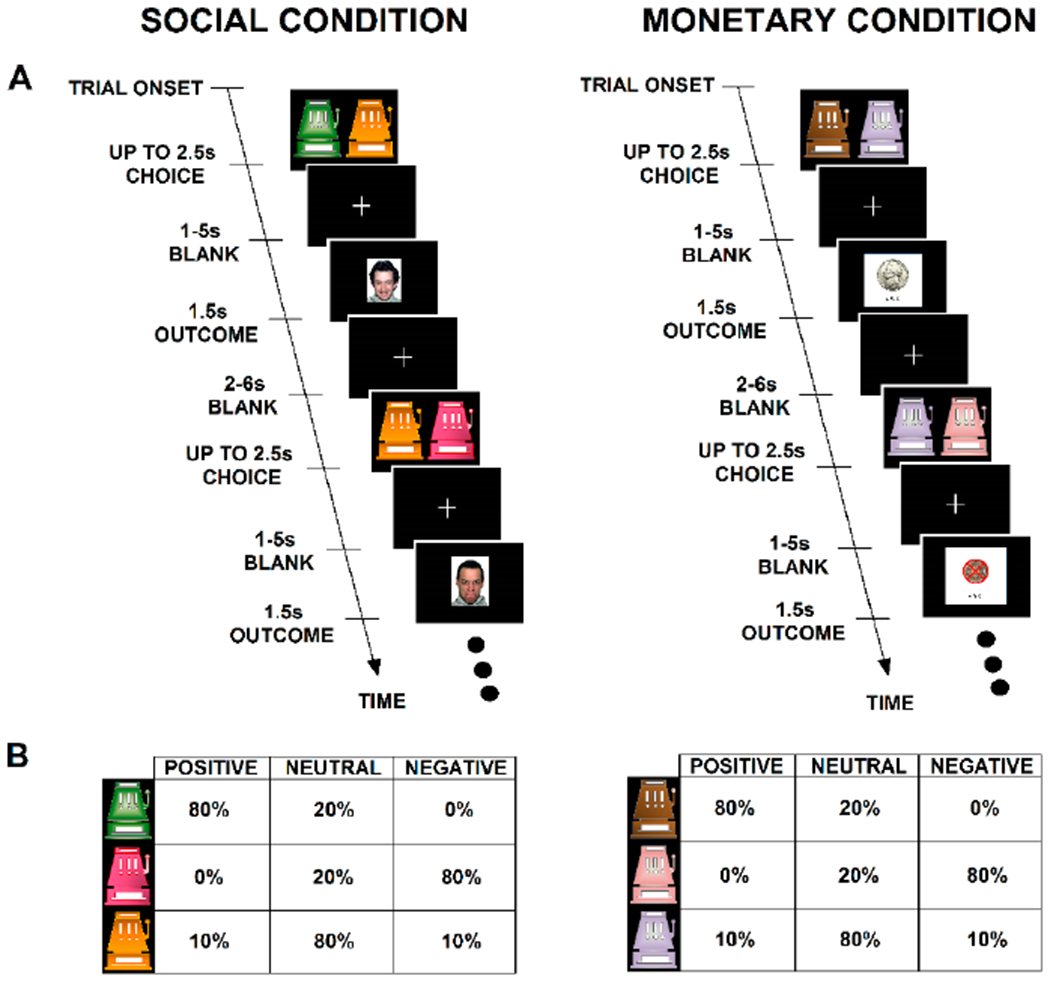
Schematic of the parallel social and monetary probabilistic reward learning tasks used in this grant (**A, B**). The tasks are based on the work of Lin and colleagues [14,33].

The PRL task [14,33] that is being used allows assessment of learning to positive or negative feedback in structurally identical social and monetary learning tasks. On each trial, there is a “choice” screen consisting of two different colored side-by-side cartoon slot machines (Figure 2A). Each slot machine color is probabilistically associated with a specific type of reward outcome (positive, negative, or neutral) 80% of the time (Figure 2B). The neutral slot machine is paired with either the positive or negative slot machine. The outcome consists of pictures of positive, negative, or neutral monetary (nickel, crossed out nickel, or blank circle) or social (happy, angry, or neutral face) feedback, respectively. Faces from four people, showing each of the three emotions from the NimStim set of faces [87], were used and were counterbalanced to include two women and two men (one black and one white for each sex) for a total of twelve stimuli. Faces are thus repeated during the task. Habituation effects of seeing the same faces more than once will be examined by looking at MRI activations to each of the three emotions in the first half of the trials compared to the second half of the trials and using trial number as a parametric modulator. There are 100 trials for both the social and monetary conditions, each of which include 50 trials in which it is possible to obtain a positive outcome and 50 trials in which it is possible to avoid a negative outcome. Participants are told they will receive the money that they win. This was also done in the original paired social and monetary PRL tasks that this grant task was adapted from [14,33] and also in monetary PRL tasks used in schizophrenia [25,31,41]. However, there are pros and cons to paying people when the monetary task is paired with a social task. A “con” is that there is an extrinsic reward for one of the tasks (monetary) but not the other (social). There are, however, several “pros”. Monetary reward may serve to motivate people on that task to perform better and also increases the ecological validity because in the “real world” if people are working for money, they are usually paid. It should also be noted that the payout was quite small (5 cents per correct response). Social rewards are generally intrinsic (e.g., effects of receiving praise or a smile) so that is also ecologically valid. A further “pro”, as described below, is that controls perform similarly on both tasks, so that they appear to be equivalently rewarding. In preliminary testing, we found that patients with schizophrenia had difficulty learning the original task to above chance criteria. Two changes were made so that people with schizophrenia could learn the task more easily. First, the outcome of the “neutral” slot machine in the original task was 33.3% neutral, 33.3% negative, and 33.3% positive which made it difficult for participants to learn that the “neutral” slot machine gave a better outcome than the “negative” slot machine. We changed the outcome of the neutral slot machine to 80% neutral, 10% positive, and 10% negative feedback. Second, we added a practice session before the MRI scan, which consisted of outcome probabilities of 90%, rather than 80% as they are during the task. Different slot machines were used in the practice than during the actual task. An additional change is that 5 cents is given for each correct response, whereas in the original task $1.00 was given for each correct response. Once these changes were made all patients in pilot work performed above chance. All controls met these criteria even before the changes were made. Pilot behavioral work showed that for controls, as desired, rewarding properties of social and monetary tasks were similar. Preliminary behavioral work from the funded grant shows that patients have impaired learning to both social and monetary reward compared to controls and that social reward learning in patients is related to negative symptoms [88].

Because people with schizophrenia have difficulty with emotion recognition [89–91], we are also administering a separate face emotion recognition test that includes the happy, angry, and neutral faces presented in the task, as well as a more complex emotion recognition task with six emotions (ER-40) [92]. Preliminary work from the funded grant shows that both patients and controls are able to accurately identify the limited set of emotions (mean ± SEM, patients’ percent correct: 98 ± 0.01; controls: 100 ± 0). Similarly, both groups rated happy faces as most pleasant, angry faces as least pleasant, and neutral faces as intermediate. Thus, between group differences in social reward learning were not attributable to impairments in facial emotion recognition [88].

To estimate trial-to-trial effects of feedback on learning, we are utilizing a Rescorla-Wagner model [93], with learning rates corresponding to positive and negative RPEs. This model assumes that participants choose probabilistically according to a *softmax* distribution, with an inverse temperature parameter connecting the trial-to-trial fluctuations in EV to the choices [94]. Using data for each trial, the BOLD response at choice is modulated by EV and the response at outcome is modulated by RPE for each participant for the social and monetary conditions [14]. MRI analyses are ongoing.

### Clinical Assessments and Questionnaires

Clinical assessments and questionnaires include: (1) Positive and Negative Syndrome Scale [95] which assesses the presence and severity of symptoms commonly found in schizophrenia; (2) Clinical Assessment Interview for Negative Symptoms [96] which is a clinician interview instrument assessing motivation/pleasure in both the social and non-social domains, and assessing expressivity. A self-report version that only includes motivation and pleasure items (MAP-SR) [97] will also be administered; (3) Chapman Social Anhedonia Scale Revised [98] which is a self-report measure of social anhedonia; (4) Chapman Physical Anhedonia Revised [99] which is a self-report measure of physical anhedonia; (5) Temporal Experience of Pleasure Scale [100,101] which is a self-report measure of anticipatory and consummatory pleasure; and (6) Specific Level of Functioning Scale [102] which includes self-report and informant assessment of social and non-social function. The informant assessment gives more reliable information so these two scales will be used separately [103,104]; and (7) Penn Emotion Recognition Task (ER-40) [92] which is a computerized test of facial emotion recognition.

### MRI

#### Image acquisition

The MRI data will be collected using a 3.0T Siemens Tim Trio system with a 32-channel head coil. A T1-weighted anatomical image will be acquired for each subject using a sagittal magnetization-prepared rapid gradient echo sequence. Functional images will be collected using a gradient echo planar sequence (TR = 1400 ms, TE = 30 ms, FA = 65°, FOV = 216 mm, 56 interleaved slices, voxel size = 2.4 mm^3^, 90 × 90 matrix, multi-band (MB) = 4, 18.01 min each for the social and money tasks). The resting-state scan will use the same parameters as the functional scan and lasts 9:35 min.

#### Image processing and MRI analyses

##### fMRI:

Images will be pre-processed using AFNI [105], including motion correction, registration to Talairach atlas space, conversion to percent signal change (for each trial type), and smoothing (6 mm FWHM Gaussian kernel). First level analyses for each task will be conducted using a GLM in which motion parameters, choice, choice modulated by EV, outcome, and outcome modulated by RPE will serve as regressors. Each trial type will be convolved with a canonical hemodynamic (gamma) response function. Beta weights for each ROI and condition will be extracted from each subject’s processed data for the second-level analyses described below (See Statistical Analyses Section). ROI analyses will be conducted using mean activation in 10 regions: bilateral vmPFC, caudate, putamen, nucleus accumbens, and amygdala. EV and RPE will be calculated as described above (Reward Learning and Face Emotion Assessment Tasks Section). vmPFC activations will be assessed at time of choice modulated by EV. Nucleus accumbens, caudate, and putamen activations modulated by RPE and unmodulated amygdala activation will all be assessed at time of outcome. Exploratory whole brain analyses will be performed to determine other brain regions that are activated by social reward learning. *Resting state:* Data will be analyzed using DPABI, Data Processing & Analysis for (Resting-State) Brain Imaging, v. 2.3 [106], which runs under Matlab. We will take great care to address issues of head micromovements, which can introduce artifact into resting state analyses [107,108]. Data will be smoothed with a 6mm full-width half-maximum kernel. Bandpass filtering will be used to retain frequencies between 0.01 Hz and 0.1 Hz. In exploratory voxelwise analyses, the functional connectivity maps from each seed region (ROIs as described for fMRI analysis) will be examined separately.

### Sample Size and Power Analysis

Reward processing studies in schizophrenia performed by Dr. Barch (a consultant on this grant) and colleagues, have found effect sizes of 0.86–1.17 in frontal areas [41,57] and 0.49–0.76 in putamen [41]. A sample size of 40 participants per group provides power of 0.80 to detect a difference between groups with a medium effect size of *d* = 0.56 (one-sided *t* test) at *p* = 0.05. Behavioral effect sizes in our preliminary data were large (e.g., Cohen’s *D* = 0.8–1.5) for behavioral reward learning differences between groups. For both MRI data and behavioral data, we have power of 0.80 to detect a difference between groups with a medium effect size of *d* = 0.56 (one-sided *t* tests), which is smaller than the effects we observed. Correlations will be performed within groups. With a sample size of 40 per group, we will be able to detect correlations of *r* = 0.38 (one-sided) with power of 0.80.

### Statistical Analyses

#### Aim 1: Assess whether people with schizophrenia show impaired learning from social rewards.

*Primary Analysis:* An MLM analysis will be carried out with fixed effects of cohort, trial type (negative and positive), and task (social or monetary) and participants as a subject effect. The primary analysis will use proportion of optimal choices in each of 10 blocks of trials. Planned comparisons will determine if proportion of optimal choices is greater in negative vs. positive trials for patients compared to controls. *Secondary analyses:* Similar analyses will be carried out using dependent variables of: (a) win-stay and lose-shift for each trial; (b) RPE for each trial; (c) EV for each trial (H1).

#### Aim 2: Evaluate neural circuitry involved in social reward learning in schizophrenia.

*Primary Analysis:* MLM analyses will be performed separately for choice (with ROIs of bilateral vmPFC modulated by EV) and outcome (with bilateral ROIs of nucleus accumbens, caudate, and putamen modulated by RPE, and amygdala) with beta weights, cohort, and task (social or monetary) as fixed effects and participants as a subject effect. Planned comparisons will determine whether activation is greater in patients than controls for each ROI (H2). *Secondary analyses:* For vmPFC modulated by EV, trial type (negative and positive) will be included in the analysis. For nucleus accumbens, caudate, and putamen modulated by RPE, and amygdala, valence (negative, positive, or neutral outcome) will be included in the analysis (H3). An exploratory goal is to assess vmPFC/striatum, mPFC/amygdala and amygdala/striatum functional connectivity (H4).

#### Aim 3: Assess relationships between social reward learning and clinical symptoms and function in schizophrenia.

*Primary Analysis:* Correlations will be carried out between the total optimal choices on the social behavioral tasks and: (a) motivation/pleasure Z score from the Clinical Assessment Interview for Negative Symptoms; (b) combined Chapman Physical and Social Anhedonia scores; and (c) informant score on the Specific Level of Functioning Scale (H5). *Secondary Analyses:* For the social and monetary reward learning tasks, correlations will be determined using separate scores from the social and non-social items from the Clinical Assessment Interview for Negative Symptoms, Chapman Scales, and Specific Level of Functioning Scale. Correlations between beta weights for the pre-planned ROIs as well as functional connectivity between pre-planned brain areas and these clinical measures will also be assessed (H6).

## SUMMARY AND FUTURE DIRECTIONS

Impaired ability to learn from social feedback may impair learning appropriate social interactions, and thus significantly contribute to functional impairment in schizophrenia. Understanding aspects of impaired social reward learning could provide an opportunity to develop more targeted social behavioral treatments. If responses to social feedback are impaired, as suggested also by several recent studies [16,19,109] this would suggest that remediations addressing social interactions and feedback from those interactions are important to further develop. Social reward learning is, of course, multiply determined, but if patients show decreased value to social rewards (i.e., decreased EV in the modeling from the current study), they may be less likely to seek them out. Remediations could also capitalize on the ability, for instance, to learn from specific types of feedback (e.g., negative social feedback) or provide impetus to train people on specific types of feedback for which they have difficulty (e.g., positive social feedback), and focus on value of social rewards. Further, if the vmPFC shows impaired EV signaling and/or connectivity, this area could be targeted for remediation with brain stimulation. In addition, reward learning is one of the Research Domain Criteria constructs, and the proposed studies will also open the way for future Research Domain Criteria studies of social reward learning using a dimensional approach. This is highly significant as people with a number of other disorders as well as people without diagnoses show impaired social interactions. This project has the potential to elucidate neural underpinnings of impaired social motivation and pleasure and provide knowledge that will enhance treatments.

Future directions thus include: (1) a larger N in an RO1 submission, which will allow us to examine both a priori brain regions and further brain areas suggested by exploratory whole brain analyses in the proposed R21, and to utilize a Research Domain Criteria approach to include other groups of participants (e.g., autism spectrum disorders and bipolar disorder) to assess specificity; (2) development of a more ecologically valid paradigm that, for instance, would involve interactions between people in a social reward paradigm; (3) examining relationships between social reward and performance on tasks that assess motivation; (4) determine psychometric properties (i.e., reliability and validity) of the social reward PRL task so that it can be developed for use as an outcome measure in remediation studies; (5) and use of results to inform novel remediation development.

## ABBREVIATIONS:

EV: expected value
MRI: magnetic resonance imaging
mPFC: medial prefrontal cortex
NKI: Nathan Kline Institute for Psychiatric Research
OFC: orbitofrontal cortex
PRL: probabilistic reward learning
RPE: reward prediction error
TPJ: temporoparietal junction
vmPFC: ventromedial prefrontal cortex
